# Molecular Characterization of Methicillin-Sensitive *Staphylococcus aureus* from the Intestinal Tracts of Adult Patients in China

**DOI:** 10.3390/pathogens11090978

**Published:** 2022-08-26

**Authors:** Yang Li, Yuanyue Tang, Zhongyi Jiang, Zhenyu Wang, Qiuchun Li, Xinan Jiao

**Affiliations:** 1 Jiangsu Key Lab of Zoonosis, Jiangsu Co-Innovation Center for Prevention and Control of Important Animal Infectious Diseases and Zoonoses, Yangzhou University, Yangzhou 225000, China; 2 Key Laboratory of Prevention and Control of Biological Hazard Factors (Animal Origin) for Agri-Food Safety and Quality, Ministry of Agriculture of China, Yangzhou University, Yangzhou 225000, China; 3 Joint International Research Laboratory of Agriculture and Agri-Product Safety, Yangzhou University, Yangzhou 225000, China

**Keywords:** methicillin-sensitive *Staphylococcus aureus* (MSSA), adult patients, intestinal tract, epidemiology

## Abstract

Intestinal infections caused by methicillin-sensitive *Staphylococcus aureus* (MSSA) have posed a great challenge for clinical treatments. In recent years, the intestinal carriage rates of MSSA have risen steadily in hospital settings in China. However, the epidemiology and molecular characteristics of MSSA from the intestinal tracts of Chinese adult patients remain unknown. In the present study, a total of 80 *S. aureus* isolates, including 64 MSSA and 16 methicillin-resistant *Staphylococcus aureus* (MRSA), were recovered from 466 fecal swabs in adult patients between 2019 and 2021 in China. The MSSA isolates exhibited high resistance to penicillin (92.2%) and erythromycin (45.3%). In addition, a higher proportion of MSSA isolates (14.1%) were multidrug-resistant (MDR) strains than that of MRSA isolates (1.3%). Among the 64 MSSA isolates, we identified 17 MLST types, of which ST398 and ST15 were the most predominant types. The most frequently detected resistance genes were *blaZ* (87.5%) and *erm(C)* (21.9%). The hemolysin genes (*hla*, *hld*, *hlgA*, *hlgB*, *hlgC*) were detected in all the MSSA isolates, but the Panton–Valentine leucocidin (*pvl)* gene was identified in 1.7% of the MSSA isolates. Our findings indicated that the prevalence and antimicrobial resistance of intestinal MSSA was a serious concern among adult patients in China.

## 1. Introduction

*Staphylococcus aureus* is a commensal bacterium in humans. It can cause various infections, such as mild soft skin and tissue infections (SSTIs), endocarditis, pneumonia and sepsis [1]. It is estimated that approximately 30% of healthy individuals carry *S. aureus* in the anterior nares [2]. 

The anterior nares are regarded as the primary site of *S. aureus* colonization [3], but increasing evidence indicates that the throat, rectum and respiratory tract are also important carriage sites for *S. aureus* [4]. Although the average prevalence of intestinal *S. aureus* carriage is approximately half of that for nasal carriage, the intestinal carriage has been shown to play a critical role in the pathogenesis of *S. aureus* infections [5]. It has been reported that intestinal and nasal carriage patients are more likely to develop *S. aureus* infections than those with nasal colonization alone [6]. The intestinal carriage can serve as a reservoir for the spread of *S. aureus* and is a potential cause of antibiotic-associated diarrhea [7]. The emergence of intestinal *S. aureus* colonization has caused a global concern regarding clinical treatments. In Europe, a higher prevalence of intestinal *S. aureus* was reported in newborns and children than in adults [5]. Similarly, an increased incidence of intestinal *S. aureus* was recorded in pediatric patients in Southern China (above 20%) [8]. However, intestinal *S. aureus* infections were infrequently reported in Chinese adult patients.

This study aimed to determine the molecular epidemiology of intestinal MSSA isolates from adult patients in Yangzhou, China. In addition, the antimicrobial susceptibility, molecular characteristics, genetic relationship, resistome and virulome features of MSSA from the intestinal tract were also investigated based on whole genome sequencing (WGS) analysis.

## 2. Results

### 2.1. Prevalence and Antimicrobial Susceptibility of Intestinal S. aureus Isolates

A total of 80 *S. aureus* and 16 MRSA isolates were detected from 466 intestinal samples. The intestinal carriage rate of MSSA and MRSA was 13.7% (64/466) and 3.4% (16/466), respectively. 

Antimicrobial susceptibility testing was performed on the confirmed 80 *S. aureus* isolates (Table 1). The antimicrobial susceptibility results for each *S. aureus* isolates are presented in Table S1. More than 93.0% of *S. aureus* isolates were resistant to penicillin, followed by 38.8% to erythromycin, 20.0% to cefoxitin and 16.3% to ciprofloxacin. All the *S. aureus* isolates were sensitive to vancomycin, rifampicin, linezolid and nitrofurantoin. Out of 80 *S. aureus* isolates from the intestinal tract, 24 (30.0%, 24/80) were resistant to only one antimicrobial agent (penicillin), and two strains YZU1686 and B11 were sensitive to all 14 tested antimicrobial agents (Table S1). Compared with MRSA isolates, the MSSA isolates in this study exhibited a higher resistance rate to erythromycin (45.3% vs. 12.5%, *p* < 0.001), ciprofloxacin (18.8% vs. 6.3%, *p* < 0.001), tetracycline (17.2% vs. 0.0%, *p* < 0.001) and sulfamethoxazole–trimethoprim (15.6% vs. 0.0%, *p* < 0.001). A total of 27 isolates, including 25 MSSA and two MRSA, showed resistance to at least three antimicrobial agents. In addition, nine intestinal MSSA isolates (14.1%, 9/64) were multidrug-resistant (MDR) strains with resistance to four or more antimicrobial agents, while only one (6.3%, 1/16) intestinal MRSA isolate was MDR. The predominant resistance phenotype of the 16 intestinal MRSA isolates was P-FOX (87.5%, 14/16) (Table S1). 

### 2.2. Molecular Typing of the S. aureus Isolates

Thirty-eight distinct spa types were identified among the 80 intestinal *S. aureus* isolates (Table S1). The predominant spa type was t377, which accounted for 8.8% (7/80) of the *S. aureus* isolates, followed by t11687 (6.3%, 5/80), t164 (6.3%, 5/80), t189 (6.3%, 5/80) and t701 (6.3%, 5/80). 

Multi-locus sequence typing (MLST) analysis grouped the 80 *S. aureus* isolates into 22 STs, including three new STs (Table S1). The most common ST types were ST398 (12.5%, 10/80) and ST630 (12.5%, 10/80), followed by ST15 (10.0%, 8/80) and ST88 (7.5%, 6/101). Five STs were found among the 16 MRSA isolates, with ST88 (37.5%, 6/16) and ST59 (31.3%, 5/16) as the predominant STs. Among the 64 MSSA isolates, ST398 (12.5%, 8/64), ST15 (12.5%, 8/64) and ST630 (10.9%, 7/64) were the most frequently observed ST types. These 22 STs were assigned to nine clone complexes (CCs), with CC1 (16.3%, 13/80), CC8 (15%,12/80), CC398 (12.5%, 10/80), CC5 (12.5%, 10/80) and CC15 (12.5%, 10/80) the frequently represented CCs (Table 2). CC1(20.3%, 13/64) and CC15 (15.6%, 10/64) were the most common CCs among the MSSA isolates. In comparison, the most abundant CCs were CC88 (37.5%, 6/16) and CC59 (25%, 4/16) in the MRSA isolates. Of note, the isolates belonging to CC1, CC15, CC7 and CC30 were found in the MSSA isolates, while the isolates belonging to CC88 and CC59 isolates were only detected in the MRSA isolates. 

### 2.3. Phylogenetic Analysis of MSSA Isolates

**Figure 1:**
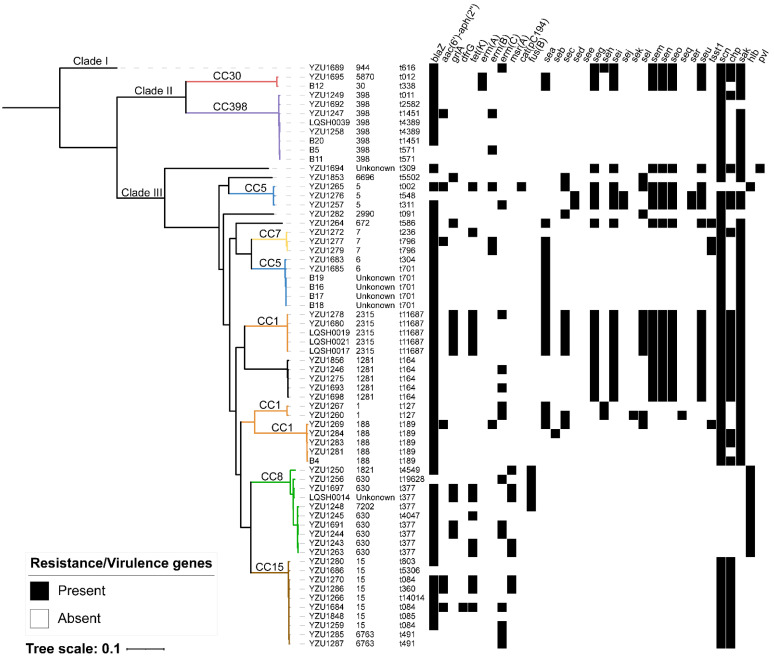
A maximum likelihood tree of the 64 MSSA genomes based on core genome SNP analysis. The information on MLST types (STs) and spa type are shown on the right of strains. The occurrence of antimicrobial resistance and virulence genes are also mapped for each strain.

The 64 MSSA isolates were subjected to whole-genome sequencing (WGS) analysis. The core-genome SNPs were underwent phylogenetic tree reconstruction using the maximum likelihood estimation (Figure 1). The results showed high genetic diversity in the intestinal MSSA isolates, which were divided into three clades (Clade I, Clade II and Clade III). Clade I contained only one isolate ST944-MSSA-t616. Clade II included ten isolates, mainly composed of CC30 and CC398 clones. Approximately 82.8% of the MSSA isolates were located in Clade III. This clade showed a more diverse genetic relationship, consisting of CC5, CC7, CC1, CC8 and CC15 clones. CC8 and CC15 clones displayed a close genetic relatedness and clustered in the same group. However, the isolates belonging to CC5 and CC1 clones were separated into different groups.

### 2.4. Antimicrobials Resistance (AMR) and Virulence Genes Analysis of the MSSA Isolates

We examined the distribution of AMR genes in the 64 MSSA isolates (Figure 1). The *β*-lactamase gene *blaZ* was the most prevalent gene detected in 87.5% of the MSSA isolates from the intestinal tract, followed by the *erm(C)* gene (21.9%), which was consistent with the results of antimicrobial susceptibility testing. The high carriage rate of the *blaZ* gene and *erm(C)* gene in the MSSA isolates may be related to the frequent use of penicillin and erythromycin in China. Among the 29 erythromycin-resistant MSSA isolates, the erythromycin resistance gene *erm(C)* (n = 14) was the most prevalent gene, followed by the *msr(A)* gene (n = 7), *the erm(B)* gene (n = 6), and *the erm(A)* gene (n = 2). In comparison, the sulfamethoxazole–trimethoprim resistance gene *dfrG* and chloramphenicol resistance gene *cat(pC194)* were detected at a low frequency of 1.6% (1/64). In addition, the aminoglycoside resistance genes were not prevalent, with 10.9% (7/64) of MSSA isolates positive for the *aac(6’)-aph(2’’)* gene. The ciprofloxacin resistance gene *grlA* and tetracycline resistance gene *tet(K)* was commonly present in the ST2315-t11687 isolates. In line with the cefoxitin-sensitive phenotypic results, no methicillin resistance gene *mecA* or *mecC* was found in all the MSSA isolates.

It has been reported that staphylococcal enterotoxins are responsible for the symptoms of food poisoning [9]. Since the MSSA isolates in this study were recovered from the intestinal tract of the adult patients with diarrhea, eighteen staphylococcal enterotoxins genes (*sea*, *seb*, *sec*, *sed*, *see*, *seg*, *seh*, *sei*, *sej*, *sek*, *sel*, *sem*, *sen*, *seo*, *seq*, *ser*, *seu* and *sep*) were tested for all the MSSA isolates. As shown in Figure 1, the *sea* gene was the most predominant staphylococcal enterotoxin in 29.7% (19/64) of the isolates, followed by the *seg* gene (28.1%, 18/64), the *sei* gene (26.6%, 18/64), the *sem* gene (28.1%, 18/64), the *sen* gene (28.1%, 18/64), the *seo* gene (28.1%, 18/64) and the *seu* gene (28.1%, 18/64). In contrast, none of the isolates carried the *see* gene. The toxic shock syndrome toxin encoding gene *tsst-1* was also detected at a low frequency (4.7%, 3/64). Fifty-three isolates harbored the immune evasion cluster (IEC) genes *scn*, *chp* and *sak*, but these genes were absent in the CC8 isolates. Additionally, the hemolysin genes (*hla*, *hld*, *hlgA*, *hlgB*, *hlgC*) were present in all the MSSA isolates, whereas the *hlb* gene was conserved in the IEC-negative isolates (Figure 1). Only one isolate (YZU1694) was found to carry the Panton–Valentine leucocidin gene *pvl*. Further analysis showed that the YZU1694 isolate contained several putative prophages including three questionable prophages (Staphy_phiSa2wa_st22, Staphy_tp310_3 and Staphy_phiPVL_CN125) and two incomplete prophages (Staphy_phiPV83, Staphy_StauST398_4), and that the *pvl* gene was located within the prophage Staphy_phiPVL_CN125.

## 3. Discussion

Intestinal colonization by *S. aureus* has been associated with an increased risk of infections and contributes to environmental contamination and disease dissemination. The prevalence of *S. aureus* in the intestinal tract of adult patients in this study was 17.2%, which was close to that detected in children (20.0%) [8], suggesting that *S. aureus* appears to easily colonize the intestinal tracts of humans. Similarly, the prevalence of intestinal *S. aureus* carriage in children from different countries was up to 23.4% [5]. It was estimated that healthy newborns exhibited a higher rate (38.5%) of *S. aureus* intestinal carriage worldwide [5]. The frequency (17.2%) of *S. aureus* in intestinal samples from adults in this study is close to previous reports in Sweden (17.0%) and Spain (15.0%) [10,11], indicating that intestinal colonization by *S. aureus* may be a reservoir for bacterial dissemination in healthcare settings [6,12]. Besides, it has also been reported that *S. aureus* can colonize the intestinal tracts of animals, such as monkeys, chimpanzees and straw-colored fruit bats [13,14,15]. Our previous study reported an intestinal carriage rate of 26.2% for *S. aureus* in monkeys in China, which is higher than that detected in humans [14]. Consistent with the previous reports of the nares as the main colonization site of *S. aureus*, our observed intestinal carriage rate (17.2%) is lower than the previously reported nasal carriage rate of 24.5% from adult patients in China [16].

In the current study, we found that 93.0% of *S. aureus* isolates were resistant to penicillin, similar to a previous report that 84.2% of *S. aureus* from children’s feces exhibited resistance to penicillin in China [8]. In contrast, a study conducted in Spain showed that only 40% of *S. aureus* from infant feces was resistant to penicillin [17]. Penicillin resistance is conferred by *β*-lactamase, which was encoded by the *blaZ* gene. The *blaZ* gene can be located on mobile elements, such as transposons, insertion sequences and plasmids [18]. However, no plasmids presented in the 64 MSSA isolates, suggesting that the *blaZ* gene is more likely to be present on the transposons or insertion sequences. All the *S. aureus* isolates were sensitive to vancomycin and linezolid; this is in agreement with previous reports of isolates from feces in other countries [11,17,19]. Traditionally, MRSA isolates display more resistance to antimicrobial agents than MSSA strains because the SCC*mec* elements in MRSA carry multiple resistance genes [20]. However, we observed that the intestinal MSSA isolates were more resistant to erythromycin, ciprofloxacin, tetracycline and sulfamethoxazole–trimethoprim than the intestinal MRSA isolates. More importantly, a higher proportion of the MSSA isolates (14.1%) were multidrug-resistant (MDR) strains, in comparison with the MRSA isolates (1.3%). These results indicated that the MDR MSSA isolates colonizing the intestinal tract may bring difficulties to further clinical treatment of patients with diarrhea or gastroenteritis. Consistent with the results of the antimicrobial-resistant phenotype, 18.8% of the intestinal MSSA isolates were found to carry the *grlA* gene, which confers resistance to ciprofloxacin [21]. Among erythromycin-resistant MSSA isolates, approximately half of the isolates carried the *erm(C)* gene. A previous report from Germany also showed that the *erm(C)* gene was responsible for erythromycin resistance in 50.7% of the 134 *S. aureus* isolates [22]. In contrast to Saribas et al., we found a low prevalence of the *erm(A)* gene [23].

The genetic diversity of ST types was detected in the intestinal *S. aureus* isolates. A total of 22 ST types were identified in the 80 isolates. The genetic diversity based on ST types was much higher for MSSA (17 STs) than for MRSA isolates (5 STs). The high genetic diversity was also detected in MSSA isolates from fecal samples of Chinese children [8]. The MSSA isolates recovered from adult patients showed a different population structure compared with the previous reported intestinal isolates from children. ST188 was reported to be the most common MSSA clone among children’s intestinal isolates in China [8]. A previous study in Spain reported that ST8 was the most common type among MSSA isolates from the feces of infants [17]. Here, ST398 and ST15 were the most predominant populations among MSSA isolates from adult patients. In European countries, ST398-MSSA has mainly been detected in swine isolates [24]. However, many studies indicate that ST398 has been characterized as the predominant molecular type of MSSA isolates from humans in China [25,26,27].

Our study showed that the frequency of the staphylococcal enterotoxin *sea* gene (29.7%), *seg* gene (28.1%), *sei* gene (28.1%), *sem* gene (28.1%), *sen* gene (28.1%), *seo* gene (28.1%) and *seu* gene (28.1%) was high in the MSSA isolates. Notably, the *seg* gene, *sei* gene, *sem* gene, *sen* gene and *seo* gene belong to the enterotoxin gene cluster (*egc*) and this cluster was reported to be located on the staphylococcal pathogenicity islands (SaPIs) SaPIn3/m3 (also known as *ν*SA*β*) [18,28]. However, a previous study conducted in Spain reported that none of *S. aureus* strains from healthy human feces harbored *egc* [11]. In addition, we observed that the *pvl*-positive rate in MSSA isolates was only 1.6% (1/64) in this study. Consistent with the low carriage rate of the *pvl* gene in this study, Benito et al. reported that none of the MSSA isolates from human feces carried the *pvl* gene [11]. We further found that the *pvl* gene in the YZU1694 isolate was located on the prophage Staphy_phiPVL_CN125. A similar result was also observed in *S. aureus* Ltr2 strain [29]. The prophages predicted in the YZU1694 isolate may serve as a reservoir for virulence genes and facilitate the spread of virulence genes to other staphylococci.

## 4. Materials and Methods

### 4.1. Sample Collection

Previous studies have defined that the culture of feces, rectal swabs and samples from the perianal area (perineum, perianal or inguinal region) can be used to screen intestinal *S. aureus* carriage [5,7]. A total of 466 fecal swab samples were collected from patients with diarrhea in Yangzhou First People’s Hospital between April 2019 and March 2021. All the swab samples were aseptically placed into sterile Whirl-Pak bags (Nasco, Fort Atkinson, WI, USA), labelled, stored on ice and immediately transported to the laboratory at Yangzhou University within 24 h.

### 4.2. Bacterial Isolation and Identification

The isolation and identification of *S. aureus* were performed as previously described with some modifications [14]. Briefly, the samples were enriched in trypticase soy broth (TSB, Beijing Land Bridge Technology Ltd., Beijing, China) containing 6.5% NaCl and incubated at 37 °C with shaking at 120 rpm for 24 h. After enrichment, approximately 10 μL of the culture was streaked onto a selective differential chromogenic agar plate (BBL CHROMagar *Staph aureus*, CHROMagar) for the selective cultivation of *S. aureus* at 37 °C for 18–24 h. The mauve colonies on this medium were regarded as suspected *S. aureus* isolates and were sub-cultured in 4 mL TSB. Genomic DNA of all the isolates was extracted using the DNeasy blood and tissue kit (Qiagen, Hilden, Germany). All isolates were identified by PCR using *nuc* primers. We then used PCR to detect the *mecA* gene in the genome to confirm the MRSA strains. 

### 4.3. Antimicrobial Susceptibility Testing

Antimicrobial susceptibility testing was carried out using the broth dilution method according to the Clinical and Laboratory Standards Institute standard from 2020 (CLSI 2020). The fourteen antimicrobial agents tested included penicillin (P), cefoxitin (FOX), vancomycin (VA), gentamicin (CN), kanamycin (K), erythromycin (E), tetracycline (TE), ciprofloxacin (CIP), nitrofurantoin (F), clindamycin (DA), linezolid (LZD), chloramphenicol (C), rifampin (RD) and Trimethoprim-trimethoprim (SXT). *Staphylococcus aureus* ATCC29213 was used for quality control. The experiment was carried out in triplicate.

### 4.4. Whole Genome Sequencing (WGS)

Genomic DNA from all the isolates was extracted using the DNeasy blood and tissue kit (Qiagen, Germany). WGS was carried out using the NovaSeq 6000 sequencing platform (Illumina Inc., San Diego, CA, USA). The sequence data of all isolates have been deposited in the NCBI under the Bioproject PRJNA838122.

### 4.5. Molecular Characteristics Analysis

WGS data were used for genotypic characterization, including spa typing and MLST. The sequence types (STs) and clone complexes (CCs) were obtained by submitting the whole genome sequence of isolates to the *S. aureus* MLST database (https://pubmlst.org/organisms/staphylococcus-aureus accessed on 15 April 2022). The spa type of each *S. aureus* isolate was determined using spaTyper (https://pubmlst.org/organisms/staphylococcus-aureus accessed on 15 April 2022). Furthermore, the WGS data were also used to identify AMR genes and virulence genes. PlasmidFinder was used to detect plasmids (https://cge.food.dtu.dk/services/PlasmidFinder/ accessed on 7 August 2022). ResFinder was used to detect AMR genes (https://cge.food.dtu.dk/services/ResFinder/ accessed on 15 April 2022). Virulence genes were identified using BLAST against the VFDB database (mgc.ac.cn/VFs/). The VirulenceFinder database was used to detect eighteen staphylococcal enterotoxins genes (https://cge.food.dtu.dk/services/VirulenceFinder/ accessed on 7 August 2022). The prophages were predicted using PHASTER [30]. The phylogenetic tree of the MRSA strains was constructed based on core genome single-nucleotide polymorphism (cgSNP) alignment using ParSNP [31]. 

### 4.6. Statistical Analysis

A χ^2^-test or Fisher’s exact test was used to analyze quantitative variables. Statistical analyses were performed using the SPSS statistical package (SPSS Inc., Chicago, IL, USA). Statistical significance was set at *p* < 0.05.

## 5. Conclusions

In conclusion, this study investigated the prevalence and molecular characteristics of MSSA isolates from the intestinal tracts of adult patients in China. In the present study, a total of 80 *S. aureus* isolates were recovered from 466 fecal swabs from adult patients in China. Our data showed that the intestinal MSSA could cause adult patient infections with a frequency of 13.7%. Out of 64 MSSA isolates, 92.2% were resistant to penicillin and 45.3% to erythromycin. More importantly, the rate of MDR MSSA isolates was 14.1%, which was higher than that of MRSA (1.3%). We determined that ST398 and ST15 were the most common types among intestinal MSSA isolates from adult patients. The *blaZ* (87.5%) and *erm(C)* (21.9%) genes were the most frequent resistance genes among MSSA isolates. All of the MSSA isolates contained the hemolysin genes (*hla*, *hld*, *hlgA*, *hlgB*, *hlgC*), while only one MSSA isolate was positive for the *pvl* gene. Therefore, it is necessary to perform continuous surveillance of MSSA in human intestinal tracts to prevent its spread in healthcare settings in China.

## Figures and Tables

**Table 1:** Antimicrobials susceptibility of S. aureus examined in this study.

Antimicrobials	MSSA (n = 64)	MRSA (n = 16)	*S. aureus* (n = 80)
Erythromycin (E)	29 ^a^ (45.3 ^b^)	2 (12.5)	31 (38.8)
Clindamycin (DA)	4 (6.3)	- ^c^	4 (5.0)
Cefoxitin (FOX)	-	16 (100.0)	16 (20.0)
Penicillin (P)	59 (92.2)	16 (100.0)	75 (93.8)
Tetracycline (TE)	11 (17.2)	-	11 (13.8)
Rifampicin (RD)	-	-	-
Linezolid (LZD)	-	-	-
Gentamicin (CN)	5 (7.8)	-	5 (6.3)
Vancomycin (VA)	-	-	-
Kanamycin (K)	9 (14.1)	1 (6.3)	10 (12.5)
Ciprofloxacin (CIP)	12 (18.8)	1 (6.3)	13 (16.3)
Nitrofurantoin (F)	-	-	-
Trimethoprim-trimethoprim (SXT)	10 (15.6)	-	10 (12.5)
Chloramphenicol (C)	1 (1.6)	-	1 (1.3)

**^a^** The number of strains that showed resistance to antimicrobial agents. **^b^** The percentage of strains with resistance to the antimicrobial agent among all the detected strains. **^c^** No strains showed resistance to the antimicrobial agent.

**Table 2:** Molecular characteristics of *S. aureus* isolates collected in this study.

CC (No.)	MLST (No.)	MSSA (No.)	MRSA (No.)	*S. aureus* (No.)
CC1 (13)	ST2315 (5), ST188 (5), ST1 (2) ST2990 (1)	13	0	13
CC8 (12)	ST630 (10), ST1821 (1), ST7202 (1)	9	3	12
CC15 (10)	ST15 (8), ST6763 (2)	10	0	10
CC5 (10)	ST5 (4), Unknown (4), ST6 (2)	9	1	10
CC398 (10)	ST398 (10),	8	2	10
CC88 (6)	ST88 (6)	0	6	6
CC59 (4)	ST59 (4)	0	4	4
CC7 (3)	ST7 (3)	3	0	3
CC30 (2)	ST30 (1), ST5870 (1)	2	0	2
Others	ST1281 (5), ST672 (1), ST944 (1), ST6696 (1), Unknown (2)	10	0	10

## Data Availability

The sequence data of all the *S. aureus* isolates have been deposited in the NCBI under the Bioproject PRJNA838122.

## Supplementary Materials

The following supporting information can be downloaded at: https://www.mdpi.com/article/10.3390/pathogens11090978/s1, Table S1: Information regarding *S. aureus* strains isolated from intestinal tract.
